# Annotating functional effects of non-coding variants in neuropsychiatric cell types by deep transfer learning

**DOI:** 10.1371/journal.pcbi.1010011

**Published:** 2022-05-16

**Authors:** Boqiao Lai, Sheng Qian, Hanwei Zhang, Siwei Zhang, Alena Kozlova, Jubao Duan, Jinbo Xu, Xin He

**Affiliations:** 1 Toyota Technological Institute at Chicago, Chicago, Illinois, United States of America; 2 Department of Human Genetics, University of Chicago, Chicago, Illinois, United States of America; 3 Center for Psychiatric Genetics, NorthShore University HealthSystem, Evanston, Illinois, United States of America; 4 Department of Psychiatry and Behavioral Neuroscience, University of Chicago, Chicago, Illinois, United States of America; University of California San Francisco, UNITED STATES

## Abstract

Genomewide association studies (GWAS) have identified a large number of loci associated with neuropsychiatric traits, however, understanding the molecular mechanisms underlying these loci remains difficult. To help prioritize causal variants and interpret their functions, computational methods have been developed to predict regulatory effects of non-coding variants. An emerging approach to variant annotation is deep learning models that predict regulatory functions from DNA sequences alone. While such models have been trained on large publicly available dataset such as ENCODE, neuropsychiatric trait-related cell types are under-represented in these datasets, thus there is an urgent need of better tools and resources to annotate variant functions in such cellular contexts. To fill this gap, we collected a large collection of neurodevelopment-related cell/tissue types, and trained deep Convolutional Neural Networks (ResNet) using such data. Furthermore, our model, called MetaChrom, borrows information from public epigenomic consortium to improve the accuracy via transfer learning. We show that MetaChrom is substantially better in predicting experimentally determined chromatin accessibility variants than popular variant annotation tools such as CADD and delta-SVM. By combining GWAS data with MetaChrom predictions, we prioritized 31 SNPs for Schizophrenia, suggesting potential risk genes and the biological contexts where they act. In summary, MetaChrom provides functional annotations of any DNA variants in the neuro-development context and the general method of MetaChrom can also be extended to other disease-related cell or tissue types.

## Author summary

A large number of genetic variants have been statistically associated with the risks of common diseases. However, whether such variants are actual risk variants and when and where they function are often unknown. To address this challenge, machine learning methods have been developed to predict functional variants in specific cellular contexts. These methods correlate DNA sequences with their biological functions, e.g. enhancer activities, and can predict effects of single base mutations. Nevertheless, the training data used by existing methods often lack neurodevelopment-related cell types, thus annotating variant effects in neuropsychiatric genetics remains difficult. In this work, we fill this gap by collecting a large set of regulatory genomic datasets from fetal and adult brain, from iPSC-based cellular models and brain organoids. We trained deep learning models on this data, and further improved its performance by borrowing information from large external datasets, a strategy known as transfer learning. Our tool, MetaChrom, is substantially better at predicting experimentally determined regulatory variants than current methods, and helps us identify candidate risk variants of Schizophrenia. We believe MetaChrom provides a valuable tool for the neuropsychiatric genetic community, and the software can be of interest to researchers in other fields as well.

## Introduction

GWAS of neuropsychiatric traits have identified hundreds of associated loci [1], however, translating these associations into detailed molecular mechanisms remain difficult. Most of variants in these loci are located in non-coding regions of the genome, with limited functional information. This makes it difficult to identify causal variants and target genes [2, 3]. In parallel to GWAS using common variants, sequencing studies also uncovered an important role of rare non-coding variants in regulatory elements in autism [4] and developmental disorders [5]. Given the low allele frequencies and reduced power of studying rare variants, having limited functional information of non-coding variations poses an even bigger problem. A key challenge in neuropsychiatric genetics is thus better annotation of functional effects of non-coding variants, ideally in a cell-type and allele-specific fashion [6].

To this end, experimental scientists have generated, in brain and neuronal cells, epigenomic maps, including chromatin accessibility and various histone marks. These data, however, usually annotate regulatory elements of hundreds of base pairs, and do not provide functional annotations at the base-level resolution. Various machine learning methods have been developed to fill in this gap. One class of methods, e.g. CADD [7], GWAVA [8], use conservation and epigenomic features, to predict likely functional variants, often based on a training set of known pathogenic variants. This approach, however, generally cannot predict allelic effects and lacks single-base resolution. More importantly, the training sets are often limited, as a result, these methods usually predict a general index of “pathogenicity” instead of context-specific effects. Another class of methods directly predict epigenomic profiles, such as protein binding sites, chromatin accessibility, histone marks and methylation, from DNA sequences [9–15]. Once trained, these models can predict the regulatory effect of a DNA variant by comparing predicted epigenomic properties of different alleles [16, 17]. These sequence-based methods can obtain single-nucleotide, and allele-specific prediction of variant effects on epigenomic features in specific cellular contexts. Because of these advantages, this approach has received great attention in the past few years [18]. In particular, deep learning based methods, such as Convolutional neural networks (CNNs), outperform traditional machine learning models in sequence-based prediction of protein-DNA/RNA interaction and chromatin profiles [16–22].

Despite these successes, it remains challenging to annotate variant effects, which often vary with cell types/tissues, in specific phenotypic contexts. Pre-trained models using public epigenomic datasets such as ENCODE and Roadmap Epigenomics Consortium [2, 23] may not include the cell types of interest and thus not able to provide the correct variant annotations in those cell types [24]. This is particularly the case for neuropsychiatric traits. Because of the difficulty of collecting samples from developing human brain, publicly available epigenomic datasets contain only a limited set of postmortem brain samples (usually adult) [25–27]. As a result, cell and tissue types relevant to early neurodevelopment, which is important for genetics of neuropsychiatric traits [25, 28, 29], are under-represented in the training set of most current variant analysis tools, making it difficult to annotate functions of variants during neurodevelopment.

We proposed to address this challenge, by collecting a large set of neurodevelopment related epigenomic datasets, while taking advantage of additional datasets with a deep transfer learning framework. We collected 31 datasets from both fetal and postmortem brains, and from cellular models of early neurodevelopment, including brain organoid and induced Pluripotent Stem Cell (iPSC) derived neuronal cells [30]. Regulatory sequences in these cellular models, as our recent work demonstrated, differ substantially from those in adult brains, and are enriched with risk variants of neuropsychiatric traits [29, 31]. Using these datasets, we trained deep Convolutional Residual Networks (ResNet). ResNet is a technique that may train very deep CNNs to enhance the predictive power, and has been proven effective in computational biology problems such as RNA binding motif discovery [32] and protein folding [33]. To further improve the performance of Resnet, we use transfer learning, a general machine learning approach that leverages knowledge and models gained from one domain to a related domain [34, 35]. Specifically, we use a CNN-based meta-feature extractor to learn rich sequence features from the 919 external epigenomic profiles of diverse cell and tissue types [2, 23], and then combine them with ResNet to learn a sequence model for the neurodevelopmental epigenomic datasets. Our strategy thus has the advantage of rich representation of ResNet, while avoiding overfitting by using sequence features learned from external datasets.

Our approached, called MetaChrom, outperforms previous deep learning methods [16] and models without transfer learning, in predicting epigenomic profiles of our data. These higher predictive accuracy translates to a better prediction of functional single nucleotide variants, as measured by their effects on chromatin accessibility. We leverage this ability of MetaChrom to annotate likely effects of variants to study genetics of schizophrenia (SCZ), a complex mental disorder. The risk of SCZ has been associated with more than 100 genetic loci via GWAS, but in most loci, the causal variants remain unknown [1]. Combining neurodevelopment-specific predictions of variant effects by MetaChrom with GWAS results, we highlight 31 likely functional Single Nucleotide Polymorphism (SNPs) in 30 SCZ-associated loci. Studying these variants points to putative causal genes in these loci and the cell types and developmental stages in which these variants likely act.

## Results

### MetaChrom: Sequence-based prediction of epigenomic profiles and variant effects using transfer learning

**Fig 1:**
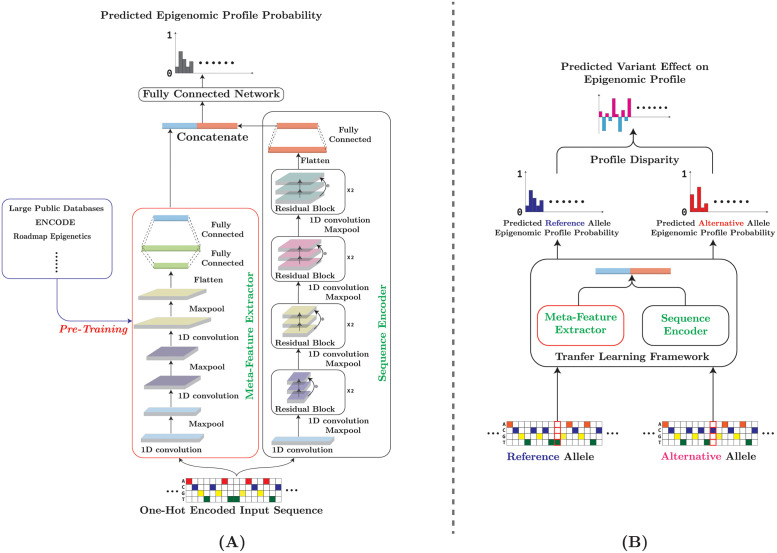
(A) Overall architecture of MetaChrom. The input sequence is fed into both MetaFeat and the ResNet sequence encoder. Their outputs are then concatenated for the prediction of epigenomic profiles.(B) Pipeline for predicting variant effect on sequence epigenomic profiles.

We have built a general deep learning model to annotate regulatory variant effects, using only DNA sequences, with limited training data. Our training set consists of a set of DNA sequences, 1000 bps in length, and their functional labels, e.g. whether a sequence is in open chromatin region or not, in a given cell/tissue type. Additionally we have access to a large compendium of publicly available epigenomic profiles—the reference epigenomic data, which will be used to extract sequence features to improve model learning capability. The modular framework we have built, MetaChrom has two major components (Fig 1A): (1) a meta-feature extractor (MetaFeat) pre-trained on the reference epigenomic data; (2) a ResNet based sequence encoder. The meta-feature and the encoded sequence are then combined to predict the epigenomic profiles of the sequence of interest. The meta-feature, i.e. transfer learning, component learns important sequence features from the reference set. While precise interpretation of sequence features in deep neural networks is generally difficult, conceptually these sequence features can be viewed as certain “regulatory code”, e.g. TF binding motifs or synergistic interaction between pairs of motifs. As shown later, learning such features would improve the model performance. Once a sequence-to-function model is trained, MetaChrom will be able to predict regulatory effects of genomic variants (Fig 1B), by comparing the predicted functional labels of two sequences differing in a single nucleotide.

### MetaChrom accurately predicts epigenomic profiles across neurodevelopment-related cell types

We applied MetaChrom to predict 31 epigenomic features, including chromatin accessibility and histone marks of enhancers, derived from both fetal and adult brain tissues or neuronal cells (See Method 4.2, Table A in S1 Table). The test sequences were obtained from chromosome 7 and 8, which were not used in the training process. We compared MetaChrom with other deep learning based methods in literature for predicting epigenomic profiles from DNA sequences. We note that the architecture of those models may depend on specific training data and it may not be easy to directly compare them with MetaChrom. For example, in the case of DeepSEA, it was designed for a large set of 919 epigenomic datasets. We thus implemented a baseline CNN model (BaseCNN) with 3 convolutional layers as representative of CNN based methods such as DeepSEA and Basset [16, 17]. We are also interested in the question of whether average epigenomic activities of a sequence across a large collection of cell types would be a good predictor of its activity in a new cell type. Such possibility has been raised in several recent papers [36, 37]. We thus obtained average epigenomic profiles, from DeepSEA, across a broad range of 919 cell and tissue types/conditions, denoted as DeepSEA-average.

**Fig 2:**
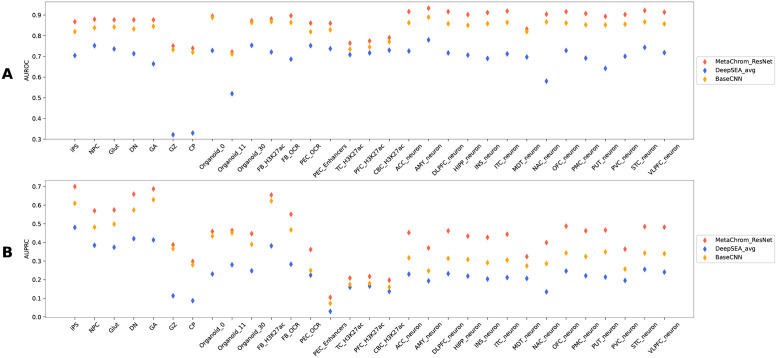
(A) AUROC and (B) AUPRC performance comparison of MetaChrom and DeepSEA method across 31 epigenomic features. NPC, Glut, DN, GA: iPSC-derived neurons. GZ, CP: germinal zone and cortical plate. OCR: open chromatin regions. See Table A in S1 Table for the list of cell/tissue types.

We evaluated the performance of these methods and MetaChrom in predicting sequence labels in the testing data using Area under Precision-Recall curve (AUPRC) and Area under Receiver Operating Characteristic (AUROC) curve. Across the 31 cell-types of interest, MetaChrom achieved higher average AUROC (0.90), and AUPRC (0.53), than the BaseCNN model and DeepSEA-average (Fig 2). Our results show the advantage of MetaChrom comparing with standard CNN and off-the-shelf tools not trained for specific cell types of interest. To give a detailed picture of how the models perform, we showed the Response-Operating Curves and Precision-Recall curves of the three methods in Amygdala neurons in Fig A in S1 Text and the complete results in Figs B and C in S1 Text.

To further investigate the importance of transfer learning and the contribution of model architecture (ResNet vs. CNN) to the performance, we performed additional comparison of MetaChrom against two variants of MetaChrom: one without transfer learning and one where CNN instead of ResNet is used. When transferred knowledge is not used, our ResNet has average AUPRC = 0.28 and average AUROC = 0.80 across 31 cell types. ResNet with the meta-feature extractor dramatically improves the performance: increasing its AUPRC from 0.28 to 0.50 and AUROC from 0.80 to 0.89, as shown in Figs D and E in S1 Text.

Our results thus highlight the advantage of the transfer learning approach of MetaChrom. We notice that our evaluation uses a large collection of 31 epigenomic features. We hypothesize that the advantage of MetaChrom over CNN would be even larger with smaller training set. This is likely a more common scenario in practice when a researcher trains a model for specific ell types of interest. To test this, we train MetaChrom and CNN on ATAC-seq data from iPSC and four types of iPSC-derived neurons. Across the five tested cell types, MetaChrom yielded AUROC of 0.87 and AUPRC of 0.82 while the average DeepSEA predictions yielded AUROC of 0.63 and AUPRC of 0.57 as shown in Fig A in S1 Text.

In summary, we demonstrate that MetaChrom is a powerful framework of predicting epigenomic profiles from DNA sequences, outperforming existing methods. Its power lies in both its ResNet architecture and its ability of transfer learning from external datasets.

### MetaChrom predicted functional variants are supported by evolutionary constraint and allelic effects on chromatin accessibility

**Fig 3:**
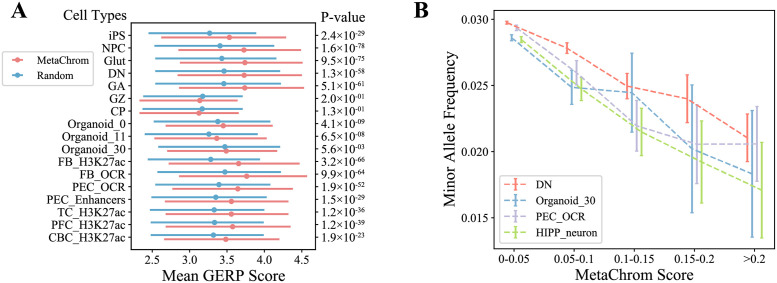
Validation of MetaChrom predicted functional variants with evolutionary constraint. (A) Distribution of GERP scores between MetaChrom predicted functional variants and random variants. (B) Minor allele frequencies of variants defined by MetaChrom scores in four selected cell types. Only variants inside peak regions of the epigenomic data were considered.

Evolutionary constraint is a commonly used metric of functional sequences [38]. We thus evaluated the accuracy of MetaChrom in predicting functional DNA variants by assessing evolutionary constraint on MetaChrom predicted variants. For all SNPs within peak regions of epigenomic data of each cell type, we computed MetaChrom scores, defined as the absolute value of the difference of MetaChrom predictions between reference and alternative alleles (Fig 1B). From these SNPs, we chose top 10,000 as predicted functional variants, and randomly sampled 100,000 variants from peak regions in the same cell type as control. We compared GERP scores, a commonly used measure of inter-species conservation [39], between predicted functional and control SNPs. In most cell types, MetaChrom top variants have significantly higher GERP scores than random ones (Fig 3A for a subset of cell types, the rest in Fig F in S1 Text), suggesting stronger evolutionary constraint. These results were confirmed with Human PhyloP scores from large 241-way mammalian alignment [40] (Fig G(A) in S1 Text). Given that functional sequences in brain may evolve relatively recently, we also assess the evolutionary constraint using only primate genomes. This analysis shows similar results (Fig G in S1 Text). We next evaluated intra-species constraint of MetaChrom variants. Because of purifying selection, functionally deleterious variants often occur at low frequencies in the population [41, 42]. We obtained minor allele frequencies (MAFs) from the gnomAD database of all variants within peak regions of 31 epigenomic profiles. We observed a clear negative correlation between MAFs and MetaChrom scores, with high scoring variants present at lower MAFs (Fig 3B for two fetal and two adult cell types, the complete results in Figs H and I in S1 Text). This results thus support the deleterious effects of MetaChrom predicted functional variants.

**Fig 4:**
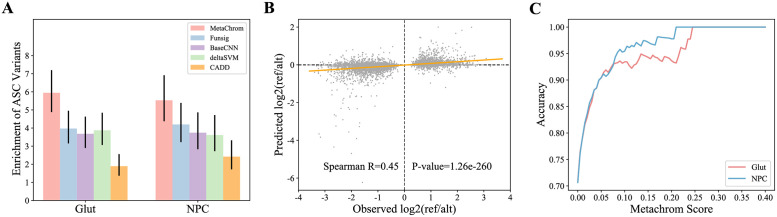
Validation of MetaChrom predicted functional variants with ASC variants. (A) Enrichment of ASC variants for predicted functional variants identified by MetaChrom, Funsig, CADD, deltaSVM, baseline CNN score in iN-Glut and NPC cells. (B) The observed allelic imbalance vs. MetaChrom predicted effects on chromatin accessibility of ASC variants in Glut neurons. (C) Accuracy of predicting directions of ASC variants in Glut and NPC cells.

To further validate MetaChrom, we compare its predictions with experimentally determined regulatory variants in iPSC-derived neurons, based on allele-specific chromatin accessibility (ASC) analysis [29]. ASC variants are defined by allelic imbalance in ATAC-seq experiments, reflecting allelic effects on chromatin accessibility and potentially gene expression. These ASC variants in iPSC-derived neurons were enriched with variants associated with gene expression, histone modification, DNA methylation, and neuropsychiatric traits [29]. We focused on ASC variants from neural progenitor cells (NPC) and glutamatergic (iN-Glut) neurons, two cell types with largest numbers of identified ASC variants. For all common single nucleotide variants (SNVs) in open chromatin regions of these two cell types, we computed their MetaChrom scores trained from the matched cell types. The top ranked 1,000 variants show about 6 fold enrichment of ASC variants, comparing with randomly sampled variants in open chromatin regions (Fig 4A). We also observed that MetaChrom scores from matched cell types generally show higher enrichment than scores from other cell types, confirming the cell type specificity of MetaChrom predictions (Fig J in S1 Text). For comparison, we also ranked variants within open chromatin regions by four other tools, including CADD, deltaSVM, FunSig, as well as a baseline CNN model trained on our collection of 31 epigenomic datasets [7, 10, 16]. CADD is widely used to predict deleteriousness of variants using a combination of evolutionary and epigenomic features. deltaSVM is a Support Vector Machine (SVM)-based supervised model for predicting variant effects, trained on the ATAC-seq data of the target cell types. Funsig is an aggregate measure of predicted regulatory effects, based on DeepSEA predictions from a large compendium of cell/tissue types (most are not from brain). Top variants by all these methods show varying levels of enrichment in ASC, but at levels lower than MetaChrom (Fig 4A). These results are robust to the number of top variants, and evaluation using Precision-Recall curve shows similar results (Fig K in S1 Text).

To test if MetaChrom can predict the effect sizes and directions of variants on chromatin accessibility, we compared the observed allelic imbalance of ASC variants in NPC and iN-Glut with the predicted differences between reference and alternative alleles. MetaChrom predictions track the observed allelic imbalance ratio with Spearman correlations of 0.45 and 0.40 in two cell types, respectively (Fig 4B and Fig L in S1 Text). Focusing on iN-Glut cells, we found 70% ASC variants show consistent signs in observed allelic imbalance and estimated effects (Fig 4B). ASC variants that are predicted to have large effects by MetaChrom show even higher agreement of predicted and observed directions of allelic imbalance. At MetaChrom score > 0.05, the agreement reaches nearly 90%, and goes even higher with higher MetaChrom score cutoff (Fig 4C). Together these results show that MetaChrom provides reasonable predictions of the regulatory effects of genetic variants.

### MetaChrom assists interpretation of GWAS results

A single locus associated with a trait from GWAS could harbor hundreds of variants in linkage disequilibrium (LD), making it difficult to distinguish causal from non-causal variants. Recent work, including our own, have demonstrated that causal signals are enriched with variants disrupting chromatin states [29, 43, 44]. Motivated by this observation, we use MetaChrom to predict functional effects and identify putative causal variants of 145 SCZ-associated loci [45] (Table B in S1 Table). We score all the common SNPs by the absolute differences of MetaChrom predictions between two alleles in each of the 31 cell types we study. Additionally, we take into account the evidence of SNPs being causal variants from previous statistical fine-mapping analysis [45]. This analysis has identified candidate SNPs at each locus, known as credible set, and quantified the evidence of individual SNPs by Posterior Inclusion Probability (PIP), Bayesian posterior probability that a SNP is a causal variant given the GWAS data. We then combine the PIP values with MetaChrom scores to prioritize putative SCZ causal variants.

**Fig 5:**
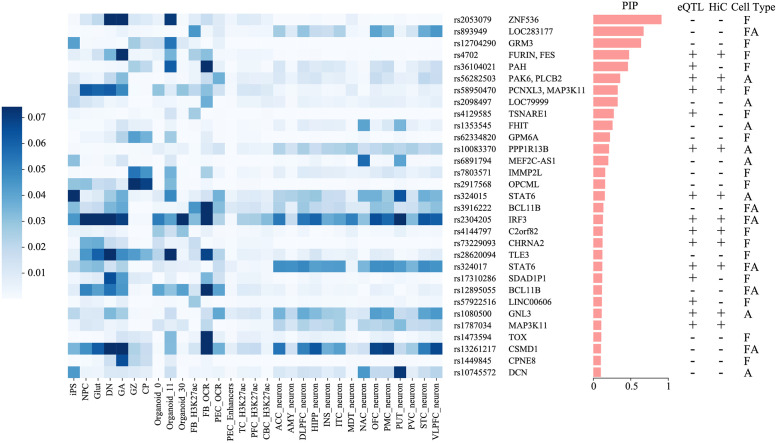
MetaChrom score of 31 candidate SNPs across 31 cell types. Candidate SNPs are ordered by their posterior inclusion probability (PIP) values shown in the middle. Three columns on the right indicate if a SNP is an eQTL (+/-), if a SNP has HiC targets (+/-) and if a SNP acts mostly in fetal stage (F) or in adult stage (A) or both (FA).

We identified 31 candidates in 30 SCZ-associated loci, based on several criteria: (i) plausibility of being SCZ risk variants (PIP > 0.1), (ii) MetaChrom scores ranked at top 1% across all common SNPs in at least one cell type, and (iii) MetaChrom scores are the very top among all SNPs in the credible set of a given SCZ risk locus, in at least one cell type (Fig 5). The list includes several high confidence SNPs with PIP > 0.5. Our results thus provide further support of the disease relevance of these SNPs, and additional information about how they may function, in terms of the relevant cell types and the epigenomic features they target. The majority of SNPs have moderate PIP values (0.1 to 0.5), and would not be considered causal SNPs by themselves. Using cell type specific MetaChrom scores, we can learn the biological context through which these variants work. Based on the cell types in which the MetaChrom score of a candidate SNP is highest among all SNPs in the credible set, we classify a SNP as acting likely in fetal stage (F) or in adult stage (A) or both (FA). Roughly equal number of SNPs in our candidates are classified as F or A, and six SNPs as FA. These findings are consistent with a recent report that expression associated variants of fetal and adult brain make comparable contributions to SCZ heritability [28]. Interestingly, once the stage (F or A) is given, the MetaChrom scores are often not very cell type specific. Most variants acting on adult stage have high scores across multiple types of adult neurons (Fig 5).

Even when causal variants are identified, their target genes may not be clear because of possible long-range regulation. To assign putative target genes, we leverage brain expression quantitative trait loci (eQTL) from post-morterm brain in GTEx [46] and CommonMinds Consortium (CMC) [47], and promoter-capture Hi-C data in iPSC-derived neurons [48]. A large fraction of our SNPs can be associated with one or more genes in eQTL, Hi-C or are located in the promoter and UTR regions. Combining these evidences and literature search, we assign the most likely target genes at each of the 31 SNPs (Fig 5 and Table C in S1 Table). Some these genes represent highly plausible risk genes of SCZ. For instance, FURIN and TSNARE1 were shown to regulate neuron growth and synaptic development by CRISPR editing in iPSC derived neurons [49]. GRM3 is a glutamate receptor and is being explored as a therapeutic target of SCZ [50]. ZNF356 is a transcription factor with an essential role in development of a subset of forebrain neurons implicated in stress and social behavior [51].

**Fig 6:**
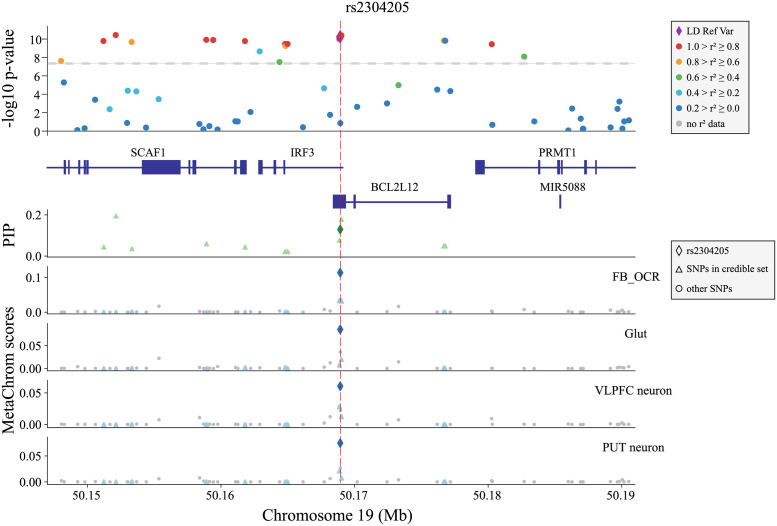
Likely causal variant rs2304205 and its MetaChrom functional annotations. The candidate SNP rs2304205 is chose as the reference variant for computing LD and it is highlighted by the red dash line in each panel. The upper panel shows significance of GWAS SNPs, LD between SNPs and genes in this region. The next panel shows credible set SNPs identified by fine-mapping (PIPs) in this region. The remaining panels show MetaChrom scores in four cell types, two in fetal stage (FB OCR and Glut) and two in adult stage (VLPEC neuron and PUT neuron).

We discuss the SNP, rs2304205, in depth to show how MetaChrom may assist the study of genetics of complex traits (Fig 6). The region containing the SNP is strongly associated with SCZ, with multiple SNPs having *p*-values below genomewide threshold (Fig 6, top). Statistical fine-mapping is insufficient to resolve the causal variant in this locus. The credible set contains 12 SNPs, but the maximum PIP is below 0.2, suggesting the uncertainty of causal variants. MetaChrom analysis highlights rs2304205 as the most plausible causal variant. It has high scores across almost all cell types, in both fetal and adult stages (Fig 5). In 24/31 cell types we examined, rs2304205 has highest MetaChrom scores among the SNPs in the credible set (see four of these cell types, two each in fetal and adult stages, in Fig 6). The SNP is located in the UTR regions of both IRF3 and BCL2L12. Only IRF3 is found as the likely target gene of rs2304205 in brain eQTL data (Table C in S1 Table). IRF3 is a key regulator of the innate immune system [52]. Recent studies show that it may be important in regulating the development of neuronal progenitor cells [53], and physically interacts with other schizophrenia susceptibility genes, such as CREB1, AKT1 and ESR1 [52]. As another example, we performed additional study of a region containing rs1080500, another SNP highlighted by MetaChrom. The SNP effect is likely limited to adult neurons and is also an eQTL in adult brain (Fig M in S1 Text). The target gene based on eQTL, GNL3, is known to regulate neuron differentiation [54]. Taken together, these case studies highlight the potential of MetaChrom in prioritization of putative causal variants.

### Identification of biologically relevant motifs from MetaChrom

To better understand what have been learned by our model, we extracted representative sequence patterns from our model using TF-MoDISco [55] and TOMTOM [56] (See Method 4.9). To focus on cell-type specific sequence features, we first sampled DNA sequence bins from our test set with mutually exclusive epigenomic feature labels. That is, each sampled sequence fragment is marked positive in only one of the 31 epigenomic features. Then we computed the gradient with respect to the input sequence for cell-type-specific saliency signals and used it as the input to TF-MoDISco. The sequence patterns generated by TF-MoDISco are then matched to known TF (transcription factor) binding motifs at human CIS-BP [57] using TOMTOM [56].

**Fig 7:**
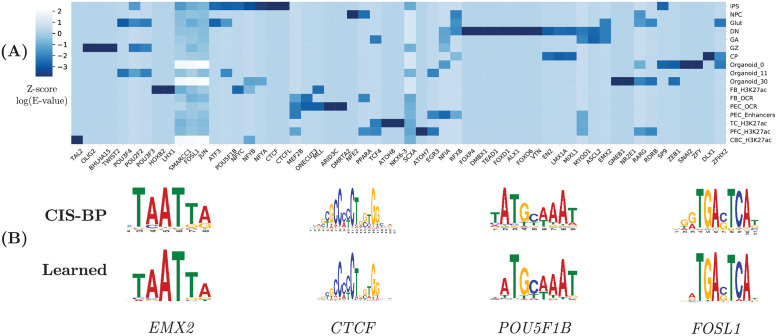
(A)Distribution of matched motifs in each epigenomic assay and (B)selected binding motifs identified by our method and their matches in the CIS-BP database.

As shown in Fig 7, our model detected known TF binding motifs specific to certain epigenomic features as well as motifs shared by multiple epigenomic features. For example, we detected binding motifs of the FOS and JUN families that form the activator protein 1 (AP-1) complex across various epigenomic features. These protein and protein complex are known to be associated with brain development [58, 59]. We detected many iPSC-specific motifs such as CTCF, an important regulator for chromatin structure [60], and POU5F1B(OCT4-PG1), a key player in the stem cell induction process [61]. In dopaminergic (DN) cell, we found motifs of proneural transcription factor FOXP4 that plays an important role in neural development [62] and FOXO6, a transcription factor closely related to cortical development [63, 64]. We discovered OLIG2, a transcription factor associated with cortical neurogenesis [65] and EN2 a transcription factor links to many stages of neural devlopment [66] in samples from the human neocortex(GZ, CP) [25].

## Discussion

Computational prediction of functional non-coding variants can facilitate the discovery and interpretation of disease risk variants. In this paper we presented a deep transfer learning method, MetaChrom, combined with a large collection of epigenomic data from brain and neuronal cells, to predict regulatory effects of base-level DNA variants. By coupling ResNet [67] with transfer learning, we show that MetaChrom is accurate in predicting neurodevelopmental epigenomic profiles. Using a combination of evolutionary constraint and experimentally determined ASC variants, we validated the utility of MetaChrom in predicting functional effects of variants. In particular, variants predicted by MetaChrom are substantially more enriched with ASC variants than the baseline CNN model and deltaSVM, two commonly used approach for annotating regulatory variants [43, 68]. We also illustrated how MetaChrom facilitates the prioritization of risk variants in GWAS loci associated with SCZ. The pre-trained models of MetaChrom are available online, and a user can query the likely functions of any variant(s) of interest using our server. We note that while the model was developed in the context of neurodevelopment, the approach and software is generic and can be applied to other user-provided epigenomic data in other biological contexts.

Many deep learning methods have been developed recently to study regulatory sequences and predict their effects. These models are generally based on CNN, e.g. DeepBind, DeepSEA, Basset, DeFine, and occasionally RNN (DanQ) [16, 17, 19, 22, 69]. It is not easy, however, to directly compare all these methods, as these models are optimized for specific training data. For instance, the popular DeepSEA method was trained on a larger set of 919 epogenomic features. For a fair comparison, we implemented an CNN model, which is currently the dominant architecture, and optimized the CNN model with our training data. As shown in our comparison, MetaChrom, by a combination of ResNet and transfer learning, outperforms the baseline CNN models in both epigenomic feature classification (Fig 2) and variant effect prediction (Fig 4).

One main application of deep learning based sequence models is the prediction of functional genetic variants. We demonstrated that MetaChrom predictions helped prioritize putative risk variants of SCZ and by combining with other datasets, revealed mechanistic insights of these variants. One limitation is that we do not yet have a single quantitative metric that combines statistical associations with deep learning based functional predictions. In our recent study [29], we show that it is possible to use ASC variants as functional information as prior in Bayesian fine-mapping. It would be interesting to extend such strategy to MetaChrom predictions. One possibility, for instance, is to combine MetaChrom predictions with experimental ATAC-seq data to have better power of detecting ASC variants. Such functionally informed genetic variant mapping has been used in eQTL studies and GWAS [70, 71]. The resulting set of deep learning-enhanced ASC set, when used as prior, may provide high resolution for fine-mapping GWAS loci.

In conclusion, we developed a deep learning based tool for predicting functional genetic variants. We demonstrated its accuracy using known regulatory variants in neuronal cells and its potential of revealing risk variants of GWAS of mental disorders. This tool is generally applicable and may enable researchers to better translate GWAS associations into mechanistic insights.

## Materials and methods

### Reference epigenomic profile data

We downloaded the epigenomic profile dataset from the DeepSEA website (http://deepsea.princeton.edu) [16]. This dataset consists of 919 chromatin features derived from the ENCODE and Roadmap Epigenomics [2, 72]. The epigenomic features are computed by first binning the reference genome (GRCh38/hg38) into 200-bp sequence fragments. The fragments were then intersected with the downloaded peaks from the public databases. Each bin was assigned a binary vector *l* ∈ *R*^*d*^(*d* = 919) as its label, each dimension representing an epigenomic feature *i* from a specific cell type with corresponding sequencing assay. If a fragment is at least 50% overlapped with the peak present in the sequencing assay *i*, the corresponding dimension in *l* is assigned 1 (i.e., *l*_*i*_ = 1), otherwise 0 (i.e., *l*_*i*_ = 0). After computing the features, the fragments were extended to 1kb to include surrounding sequences [16, 73]. All the fragments were then split into training, validation, and test sets such that all fragments from chromosomes 7 and 8 are held out for test, and the rest are randomly split into training and validation sets. The training set we used contains 4,400,000 sequence fragments with at least one positive chromatin feature. We have trained our meta-feature extractor (MetaFeat) on this chromosomal-based split so we can fine-tune the feature extractor using the test sequence. We also employed the same chromosomal-based split in our case study for neurodevelopment related tissues data(Table A in S1 Table) which ensures the test sequences are never seen by our model before test time to avoid potential training bias.

### Epigenomic profiles from neurodevelopment related tissues and cell types

To comprehensively capture the epigenomics landscape of the human neurons and brain, we collected data from 31 different epigenomic assays, from the early developmental stages to fully developed adult brain tissues. For the early developmental stages, we obtained a set of ATAC-seq peaks from iPSC derived neuronal cells described in [29], with a total of five different cell types. These cell types are good models of neurodevelopment. We also obtained one fetal brain DNase-seq sample from the Roadmap Epigenomics Project [23]; chromatin accessibility data from brain organoid samples at three different time points [74]; ATAC-seq profiles from two early human neocortex samples in germinal zone (GZ) and cortical plate (CP) [25]; and one fetal brain H3K27ac profile from [75]. For the adult brain, we collected fourteen neuronal ATAC-seq profiles from the BOCA project [76]; and five chromatin and histone features from the PsychENCODE project [77].

We processed the peaks from each epigenomic profile in a similar way as the aforementioned reference epigenomic profile data. In our dataset, each dimension in the label *l* ∈ *R*^31^ represents if a segment is active or not in a given epigenomic profile, i.e., if it is at least 50% overlapped with the peaks in each epigenomic assay. We choose our test set such that all fragments from chromosomes 7 and 8 are held-out when the rest of the genome are randomly split for training and validation. After processing, we obtained 3,165,290 sequence fragments that’s active in at least one epigenomic assay for training and validation; our test set contains 390,380 sequence fragments for model evaluation.

### MetaChrom model architecture

MetaChrom as shown in Fig 1A has two major modules: 1) a CNN-based meta-feature extractor pre-trained on a large public dataset(MetaFeat). 2) a ResNet-based sequence model that learns cell-type-specific features directly from the input sequence. To predict the regulatory profile of a DNA sequence fragment *i* of 1kbp in length, we first encode the sequence fragment as a one-hot matrix and fed it into the meta-feature extractor, which will output a vector representation $F_{meta}$ of the input sequence while the sequence is also fed to the ResNet-based sequence encoder simultaneously to obtain a cell-type-specific feature representation $F_{seq}$. The two feature representations are concatenated to form a new joint vector representation $F_{joint}$, which is fed into a fully connected dense network to predict epigenomic features $A\in R^{n_{out}}$, Where *n*_*out*_ is the number of epigenomic features of interest.

MetaFeat is a CNN model consisting of three 1-D convolutional layers with kernel length 7 and channel sizes 320, 480 and 960, respectively. This feature extractor takes a one-hot encoded sequence (of dimension 1000 × 4) as input and outputs a vector representation(*R*^919^) of the input sequence fragment. Each convolutional layer in this feature extractor is followed by a ReLU [78] layer for activation and a max-pooling layer with a kernel size of 4 for down-sampling. We used a smaller kernel size (7), while previous methods use a large kernel size (19) for model interpretability [17], but it has been shown that with appropriate interpretation tools [37, 55] meaningful binding motifs may be detected with a smaller kernel. The final convolutional layer connects to two fully connected layers, which in turn generate a vector of 919 chromatin features to represent the input.

Our ResNet-based sequence encoder maps the input sequence (encoded as a one-hot matrix of dimension 1000 × 4) to a cell-type-specific representation, which is a vector of 31 elements. The ResNet model consists of one 1D convolutional layer and eight residual blocks. The 1D convolutional layer has a kernel length 4 and channel size 48. Each residual block contains two 1D convolutional layers, each followed by a ReLU activation layer. The eight residual blocks have channel sizes of 96, 96 128, 128 256, 256, 512 and 512, respectively and kernel length 7. Finally, the last residual block connects to two fully connected layers, which generate the cell-type-specific representation for the input sequence segment.

The outputs from MetaFeat and the sequence encoder are concatenated to form a integrative vector representation of the input sequence, which is then fed into three fully connected layers to predict the probabilities of epigenomic profiles.

### Other methods for comparison

To compare our proposed method with other alternatives in the community, we implemented a baseline CNN model(BaseCNN) with 3 convolutional layers [16] and trained the models on our curated dataset for comparison. We also compared our predictions with the mean epogenomic profile predicted by DeepSEA(DeepSEA_avg) for each test sequence which was directly obtained from their server [16]. CADD scores and funsig scores are downloaded from the respective server [7, 16]. See Fig L in S1 Text for the details.

### Predicting variant effects on regulatory profiles by MetaChrom

To predict the variant effect on a given sequence’s epigenomic profile, two sequences of length 1kb differing only at and centered at the variant position are used. One of them corresponds to the reference allele and the other corresponds to the alternative allele. As shown in Fig 1B, we pass those two sequences into MetaChrom separately to predict their epigenomic profiles as *A*_*ref*_ and *A*_*alt*_. Then we compare the predicted regulator profile and compute the disparity as absolute difference |*A*_*ref*_ − *A*_*alt*_| or log odds ratio $|\text{log}\left(\frac{A_{ref}}{1-A_{ref}}\right)-\text{log}\left(\frac{A_{alt}}{1-A_{alt}}\right)|$ for measuring variant effects.

### Model training and testing

We trained our transfer learning framework in two phases. In phase one we train the meta-feature extractor on the public epigenomic profile data (see Data section) using binary cross-entropy as the loss function and the Adam optimizer [79]. In phase two we jointly train the ResNet model and the meta-feature extractor [35] with stochastic gradient descent(SGD) accelerated by Nesterov momentum [80], starting from the trained feature extractor in the first phase with binary cross-entropy loss function on the targeted cellular context data. In both phases, sequence bins from chromosome 7 and 8 are held-out for test and the training/validation sets are randomly split from the remaining bins. For model selection, we searched batch sizes of (16, 32, 64, 128, 256), ten learning rates in (1e-5, 1e-3) equally divided in log scaled distance, as well as kernel sizes of (4,6,8) for the pre-training phase. For the joint training phrase, we searched on different batch sizes of (16, 32, 64, 128, 256), learning rates in (1e-5, 1e-3) equally divided with log scaled distance. We also tested different learning decay rates and Nesterov momentum. All models are trained on an NVIDIA 2080Ti GPU in 5 hours.

### Assessing evolutionary constraint on MetaChrom predicted functional variants

A variant is scored by MetaChrom using the absolute value of the difference in MetaChrom output between reference and alternative alleles. The score ranges from 0 to 1. For top MetaChrom predicted variants in 31 cell types, we calculated and compared their GERP scores and PhyloP scores [39] with control variants, chosen randomly in peak regions of the same cell types. GERP scores were obtained from ANNOVAR [81], PhyloP scores were obtained from https://cglgenomics.ucsc.edu/data/cactus/ and http://hgdownload.cse.ucsc.edu/goldenpath/hg38/phyloP17way/, and were compared between MetaChrom predicted and control variants using the Wilcoxon Rank-Sum Test.

Minor allele frequencies (MAFs) of all variants were obtained from the Genome Aggregation Database (gnomAD) [82] using ANNOVAR. We split variants into two sets of 5 bins based on the MetaChrom score: 0–0.05, 0.05–0.1, 0.1–0.15, 0.15–0.2, 0.2–1.0 and 0–0.05, 0.01–0.02, 0.02–0.03, 0.03–0.04, 0.04–1.0. In each bin, mean MAFs were calculated to investigate the correlation between MAFs and MetaChrom scores.

### Evaluation of MetaChrom prediction using ASC variants

We used a recently published dataset of allele-specific chromatin accessibility (ASC) variants to compare several methods for predicting functional variants [29]. ASC variants are defined by allele imbalance in read counts from ATAC-seq experiments. A total of 5,611 and 3,547 ASC SNPs, at FDR < 0.05, were identified in neural progenitor cells (NPC) and gutamatergic neurons (iN-Glut), respectively.

To identify putative functional variants, we limit to single nucleotide variants (SNVs) in open chromatin regions in 2 cell types. The SNVs are retrieved from the 1000 Genomes Project with MAF > 5% [83].

We calculated scores of all these SNVs from several tools. Funsig scores were obtained from the DeepSEA Server [16] and CADD scores [7] were obtained from ANNOVAR. For deltaSVM [10, 84–86], we followed the training strategy from Shigaki et al [87]. Specifically, we trained gkm-SVM models on 300 bp sequences centered on ATAC-seq data of iN-Glut and NPC cells with LS-GKM. We set parameters -l(word length) to 11, -k(number of informative column) to 7, -d(maximum number of mismatches to consider) to 3, -t(kernal function) to 2, and other parameters follow default values. deltaSVM scores were then obtained with the trained gkm-SVM model and script deltasvm.pl. Software and scripts for deltaSVM can be found in http://www.beerlab.org/deltasvm/. Baseline CNN scores were obtained from the baseline CNN model that were trained on 31 epigenomic profiles. We chose the top 1,000 variants ranked by MetaChrom, Funsig, deltaSVM, CADD, baseline CNN score in descending order as predicted functional variants for each method. We then counted the number of ASC variants in predicted functional variants vs. control variants. The enrichment of ASC variants is then calculated by Fisher Exact Test.

In the analysis involving effect size and direction of SNPs, we define the observed allelic imbalance as log(*R*_*ref*_/*R*_*alt*_), where *R*_*ref*_ and *R*_*alt*_ denote the number of reads mapped to the two alleles, and the MetaChrom predicted effects on chromatin accessibility from our model as log(*A*_*ref*_/*A*_*alt*_). Correlation between observed allelic imbalance and MetaChrom predicted effects on chromatin accessibility is calculated by Spearman’s rank correlation coefficient.

We also estimated the percent of MetaChrom predicted variants are actually experimentally determined ASC variants. For iN-Glut cells, among top 1000 MetaChrom variants, 462 SNPs are evaluated for allele imbalance test (not all SNPs are heterozygous in the study), and 123 (27%) are reported as ASC variants. For NPC, 445 SNPs, among top 1000, are evaluated for allele imbalance test and 87 (20%) are ASC variants.

### Motif identification and visualization

Our model learns context-specific sequence patterns to predict epigenomic profiles. Many of these patterns may correspond to cell-type-specific transcription factor (TF) binding motifs. One way to interpret the models is to extract sequence patterns (motifs) from the filters of the first convolutional layer [17, 19, 69], but this strategy often yields patterns that are hard to interpret. This is because CNNs learn a distributed representation of sequence motifs and thus, an individual filter may correspond to only a partial motif that cannot be easily identified [55, 88]. Some methods assess the importance of each position in a given sequence to measure individual nucleotide contribution [89], but they do not yield interpretable motifs directly.

To extract meaningful sequence motifs from our deep model, here we used the recently-developed tool TF-MoDISco [55] that combines position-wise single-nucleotide contribution scores to generate cell-type-specific sequence patterns. We then use TOMTOM [56] to match the identified sequence patterns to known TF binding motifs in the CIS-BP database for further analysis [57]. To apply TF-MoDISco, we first randomly select 2,000 DNA bins from our testing set with mutually exclusive epigenomic features, in which each sequence bin is only marked active in one epigenomic feature but not others. Then we generate position-wise contribution score with saliency map [90], which is the gradient of the output with respect to the one-hot encoded input. The gradient is then gated by the observed nucleotide to generate importance score, i.e., only the gradient of the observed nucleotide is kept and the gradient of unobserved nucleotide is set to 0. The importance score is fed into TF-MoDISco to generate predictive sequence patterns, which were searched against the human CIS-BP [57] database for TF binding motifs using TOMTOM [56] with E-value = 1e-4.

## Supporting information

S1 Text **Fig A**. ROC and PRC plot for Amygdala neurons across different tested models. **Fig B**. (i) Average ROC and PRC plot for 31 epigenomic features across different tested models. (ii) Average ROC and PRC plot for five iPSC-derived neuronal cell types. The Average PRC performance appears better for the five cell-types model than the 31-cell-types model because some of the other cell types in the 31-cell-types model have low AUPRC values which resulted in lower average AUPRC. **Fig C**. ROC and PRC plot for (A) CNN, (B) ResNet, (C) MetaFeat-CNN, (D) MetaFeat-ResNet models on 31 epigenomic features. **Fig D**. Average ROC and PRC plot for 31 epigenomic features across different tested models. CNNBase and ResNet are baseline CNN and ResNet models without transfer learning. MetaFeat-CNN: CNN model with transfer learning. **Fig E**. (A) AUROC and (B) AUPRC performance comparison of MetaChrom and other methods across 31 epigenomic features. See Table A in S1 Table for the list of cell/tissue types. **Fig F**. Distribution of GERP scores between MetaChrom predicted functional variants and random variants sampled from the peak regions in each cell type. P-values testing the difference were computed from Wilcoxon Rank-Sum Test. **Fig G**. Evolutionary constraint evaluated by Human PhyloP scores (A) 241-way mammalian alignment from the Zoonomia Project (B) 17-way primate specific alignment. **Fig H**. Minor allele frequencies of variants defined by MetaChrom scores in 10 epigenomic profiles in fetal brain cell types. Only variants within peak regions of the data were considered. **Fig I**. Minor allele frequencies of variants defined by MetaChrom scores in 17 epigenomic profiles in adult brain cell types. Only variants within peak regions of the data were considered. **Fig J**. Number of experimentally determined ASC variants (two cell types, Glut—left and NPC—right) in top 10,000 MetaChrom predicted functional variants across 31 cell types. **Fig K**. Comparison of methods in predicting ASC variants (A) Zoomed-in Precision-Recall (PR) curve with Recall in [0,0.2] and Precision in [0,0.25]. (B) Number of ASC variants in top K prioritized variants. **Fig L**. The observed allelic imbalance vs. MetaChrom predicted effects on chromatin accessibility of ASC variants in NPC neurons. **Fig M**. Likely causal variant rs1080500 and its MetaChrom functional annotations. The candidate SNP rs1080500 is chosen as the reference variant for computing LD and it is highlighted by the red dash line in each panel. The upper panel shows the significance of GWAS SNPs, LD between SNPs and genes in this region. The next panel shows credible set SNPs identified by fine-mapping (PIPs) in this region. The remaining panels show MetaChrom scores in four cell types, two in the fetal stage (FB_OCR and Glut) and two in the adult stage (VLPFC neuron and OFC neuron). **Fig N**. Baseline CNN model Architecture.
(DOCX)

S1 Table **Table A**. Cell type information. **Table B**. Creditable SNPs set. **Table C**. GWAS Candidate SNPs.
(XLSX)
