# Supplementary material for: Annotating functional effects of non-coding variants in neuropsychiatric cell types by deep transfer learning
Source: PLoS Comput Biol. 2022 May 16;18(5):e1010011. doi: 10.1371/journal.pcbi.1010011 (PMC9135341; doi:10.1371/journal.pcbi.1010011)
Supplement: S1 Text — Fig A. ROC and PRC plot for Amygdala neurons across different tested models. Fig B. (i) Average ROC and PRC plot for 31 epigenomic features across different tested models. (ii) Average ROC and PRC plot for five iPSC-derived neuronal cell types. The Average PRC performance appears better for the five cell-types model than the 31-cell-types model because some of the other cell types in the 31-cell-types model have low AUPRC values which resulted in lower average AUPRC. Fig C. ROC and PRC plot for (A) CNN, (B) ResNet, (C) MetaFeat-CNN, (D) MetaFeat-ResNet models on 31 epigenomic features. Fig D. Average ROC and PRC plot for 31 epigenomic features across different tested models. CNNBase and ResNet are baseline CNN and ResNet models without transfer learning. MetaFeat-CNN: CNN model with transfer learning. Fig E. (A) AUROC and (B) AUPRC performance comparison of MetaChrom and other methods across 31 epigenomic features. See Table A in S1 Table for the list of cell/tissue types. Fig F. Distribution of GERP scores between MetaChrom predicted functional variants and random variants sampled from the peak regions in each cell type. P-values testing the difference were computed from Wilcoxon Rank-Sum Test. Fig G. Evolutionary constraint evaluated by Human PhyloP scores (A) 241-way mammalian alignment from the Zoonomia Project (B) 17-way primate specific alignment. Fig H. Minor allele frequencies of variants defined by MetaChrom scores in 10 epigenomic profiles in fetal brain cell types. Only variants within peak regions of the data were considered. Fig I. Minor allele frequencies of variants defined by MetaChrom scores in 17 epigenomic profiles in adult brain cell types. Only variants within peak regions of the data were considered. Fig J. Number of experimentally determined ASC variants (two cell types, Glut—left and NPC—right) in top 10,000 MetaChrom predicted functional variants across 31 cell types. Fig K. Comparison of methods in predicting ASC variants (A) Zoomed-in Prec [file pcbi.1010011.s001.docx]

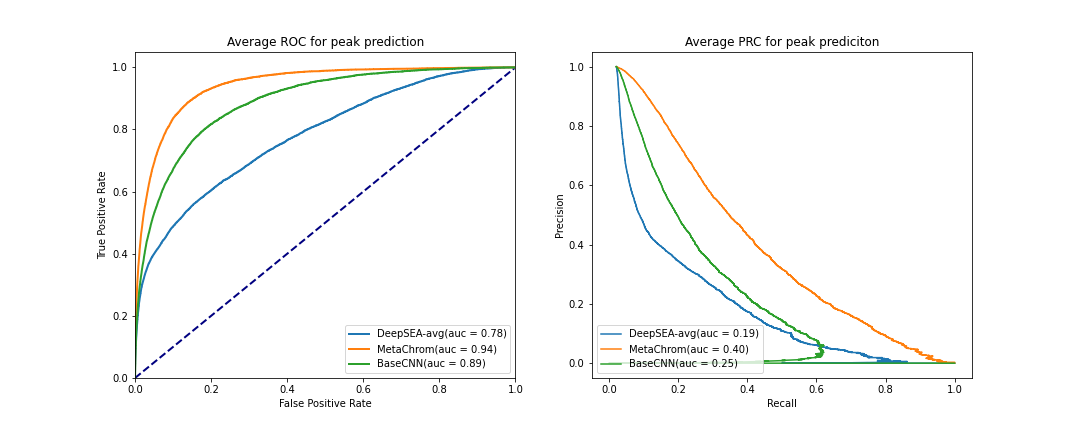


Fig A. ROC and PRC plot for Amygdala neurons across different tested models.

(i)


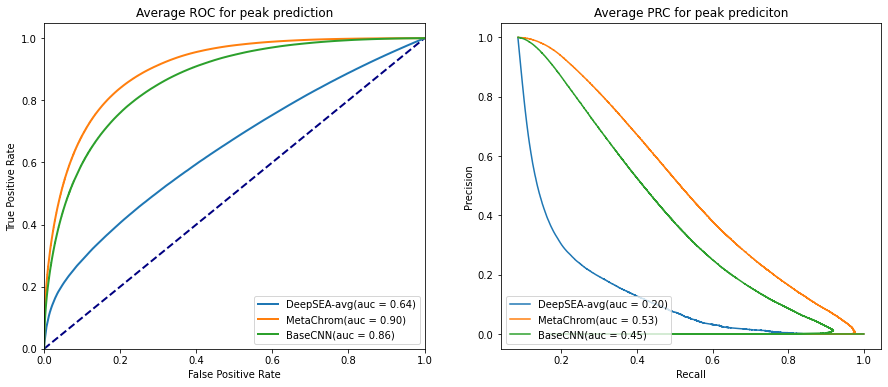


(ii)


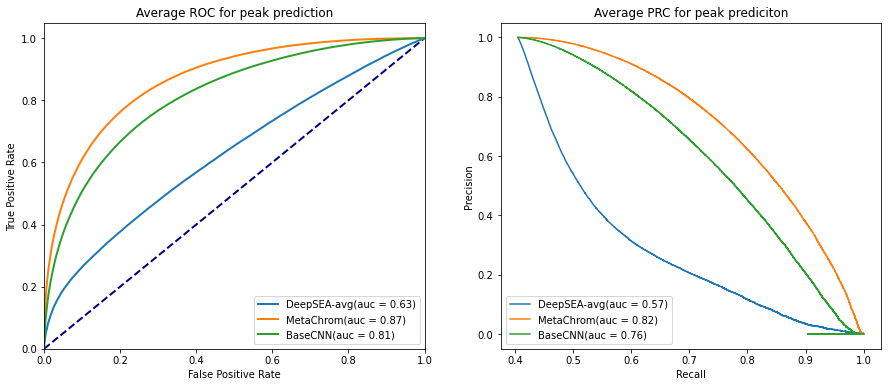


Fig B. (i) Average ROC and PRC plot for 31 epigenomic features across different tested models. (ii) Average ROC and PRC plot for five iPSC-derived neuronal cell types. The Average PRC performance appears better for the five cell-types model than the 31-cell-types model because some of the other cell types in the 31-cell-types model have low AUPRC values which resulted in lower average AUPRC. Please see Fig S3 for more information.


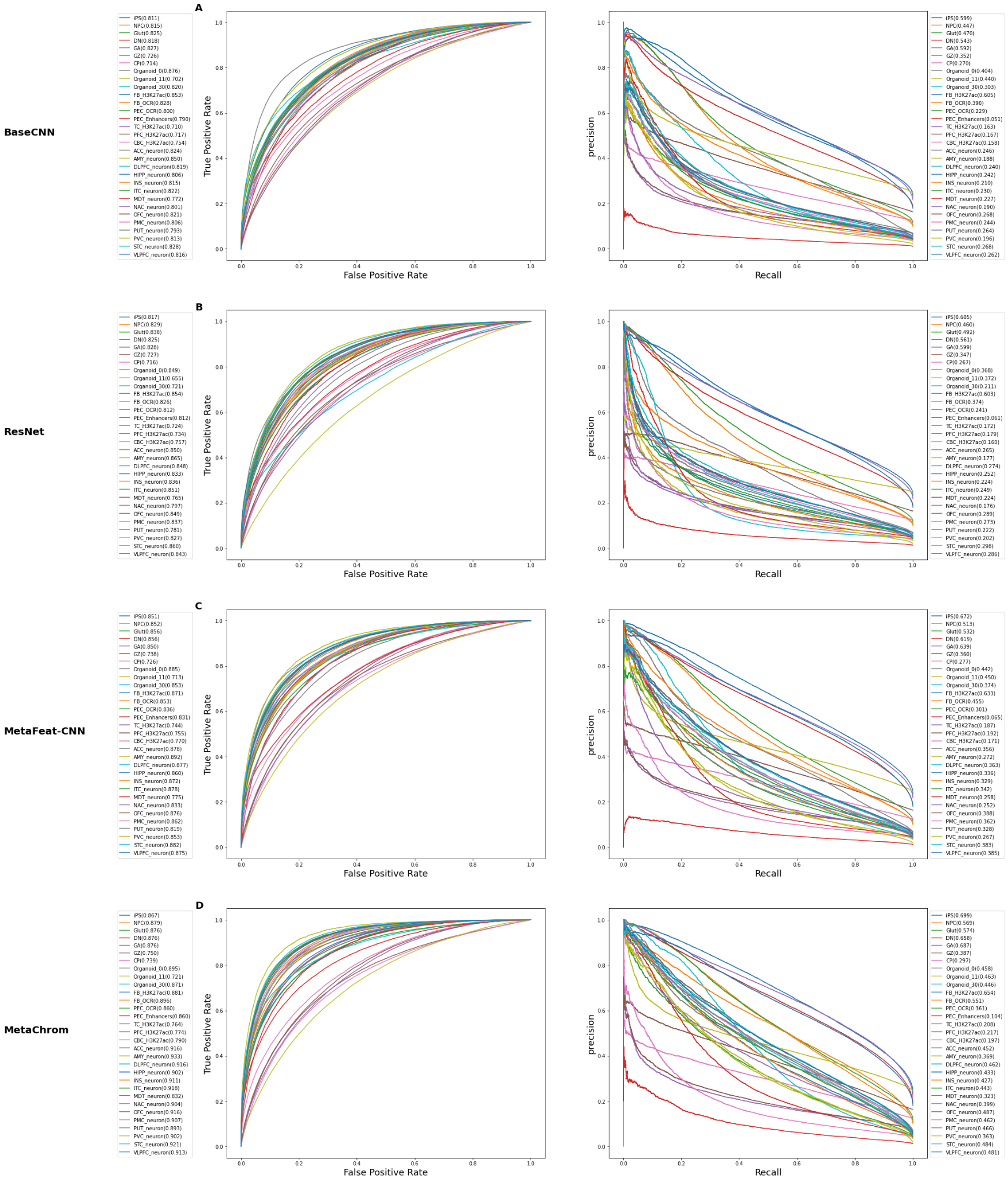


Fig C. ROC and PRC plot for (A) CNN, (B) ResNet, (C) MetaFeat-CNN, (D) MetaFeat-ResNet models on 31 epigenomic features(Method).


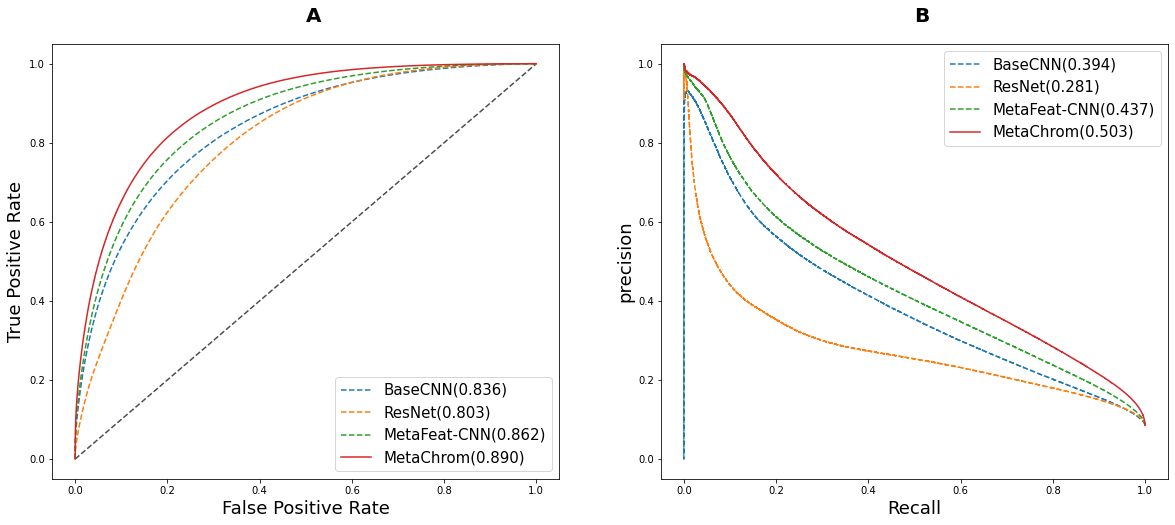


Fig D. Average ROC and PRC plot for 31 epigenomic features across different tested models. CNNBase and ResNet are baseline CNN and ResNet models without transfer learning. MetaFeat-CNN: CNN model with transfer learning.


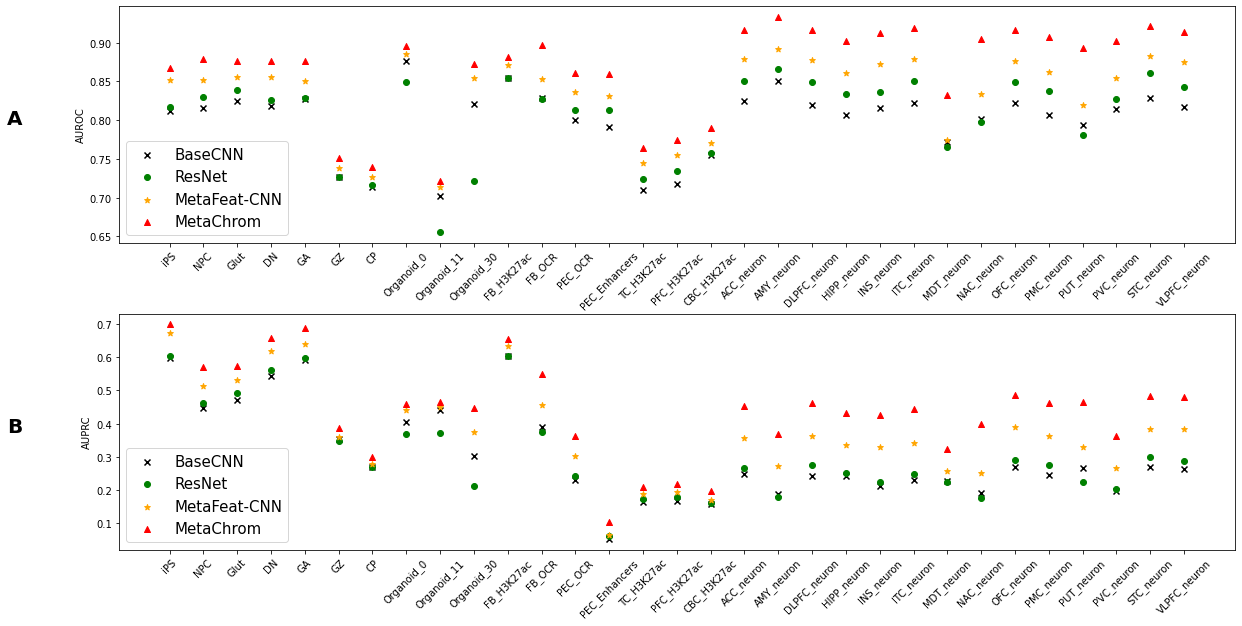


Fig E. (A) AUROC and (B) AUPRC performance comparison of MetaChrom and other methods across 31 epigenomic features. See Table S1 for the list of cell/tissue types.


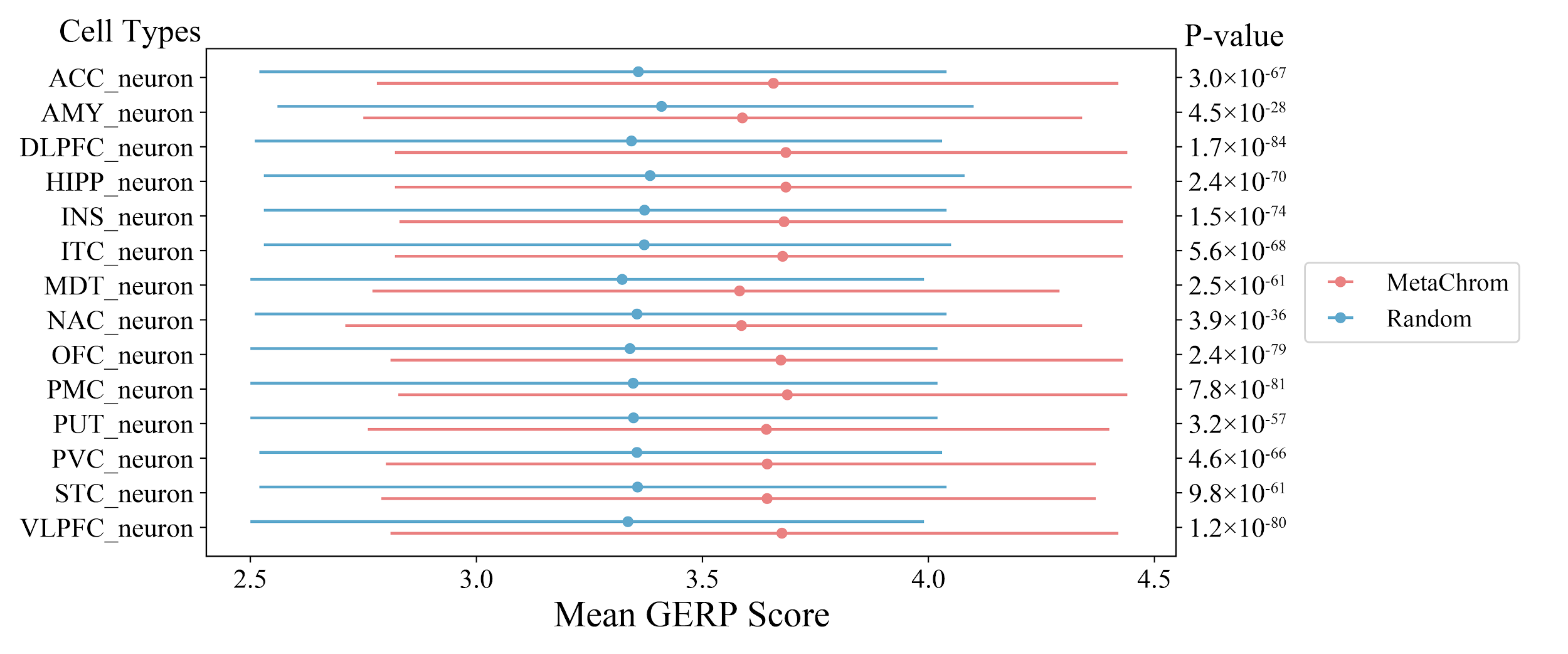


Fig F. Distribution of GERP scores between MetaChrom predicted functional variants and random variants sampled from the peak regions in each cell type. P-values testing the difference were computed from Wilcoxon Rank-Sum Test.


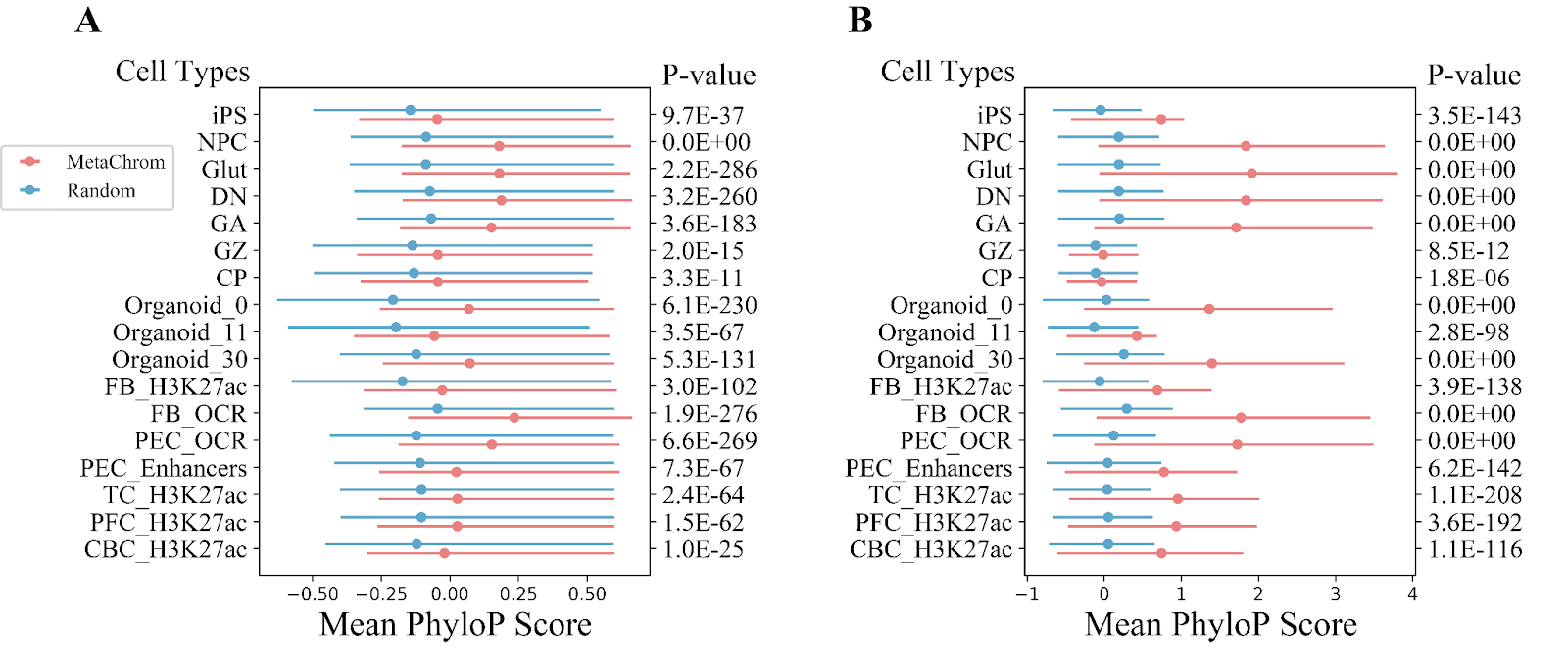


Fig G. Evolutionary constraint evaluated by Human PhyloP scores (A) 241-way mammalian alignment from the Zoonomia Project (B) 17-way primate specific alignment.


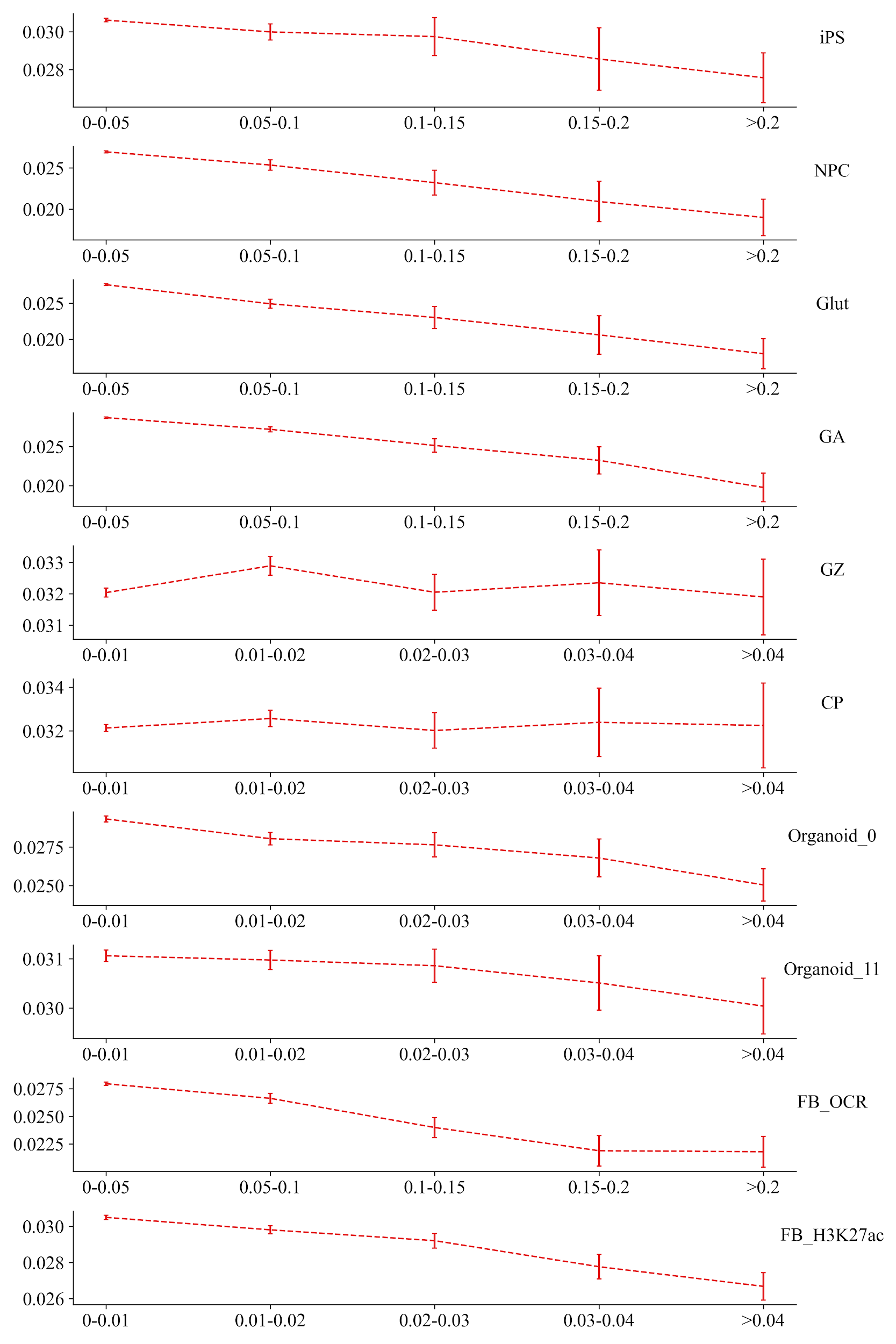


Fig H. Minor allele frequencies of variants defined by MetaChrom scores in 10 epigenomic profiles in fetal brain cell types. Only variants within peak regions of the data were considered.


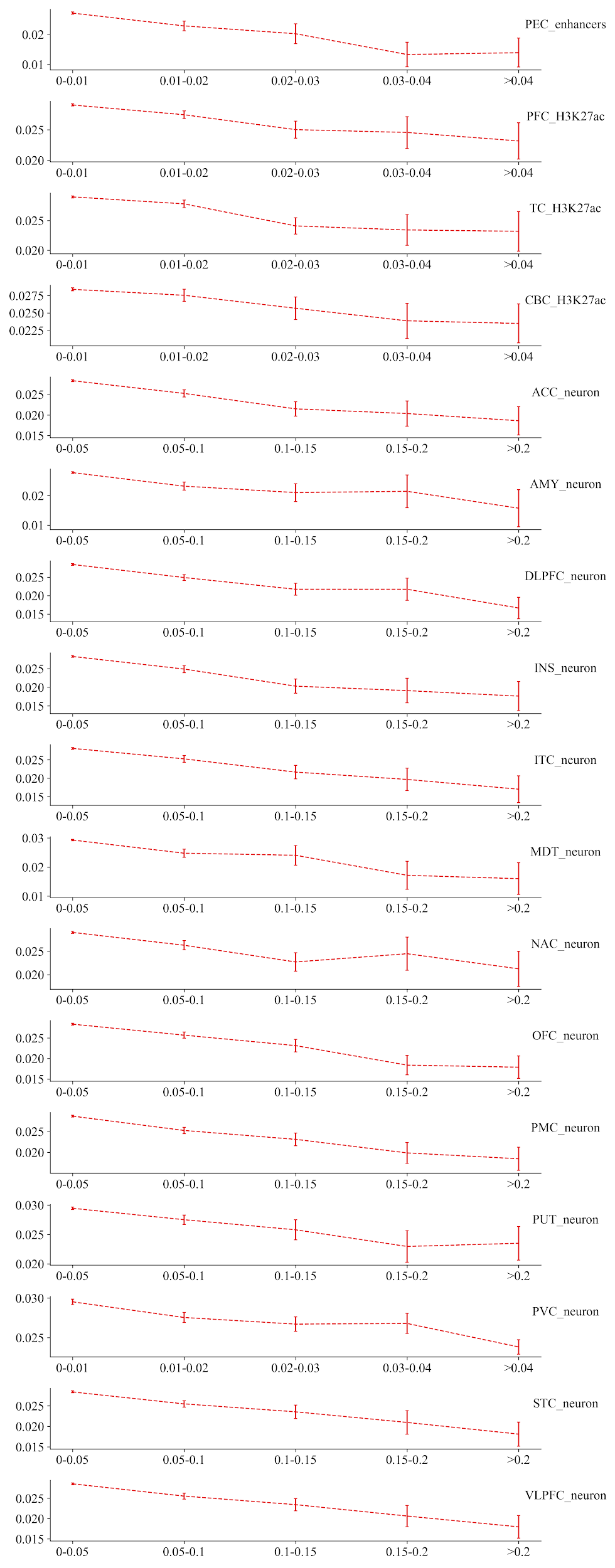


Fig I: Minor allele frequencies of variants defined by MetaChrom scores in 17 epigenomic profiles in adult brain cell types. Only variants within peak regions of the data were considered.


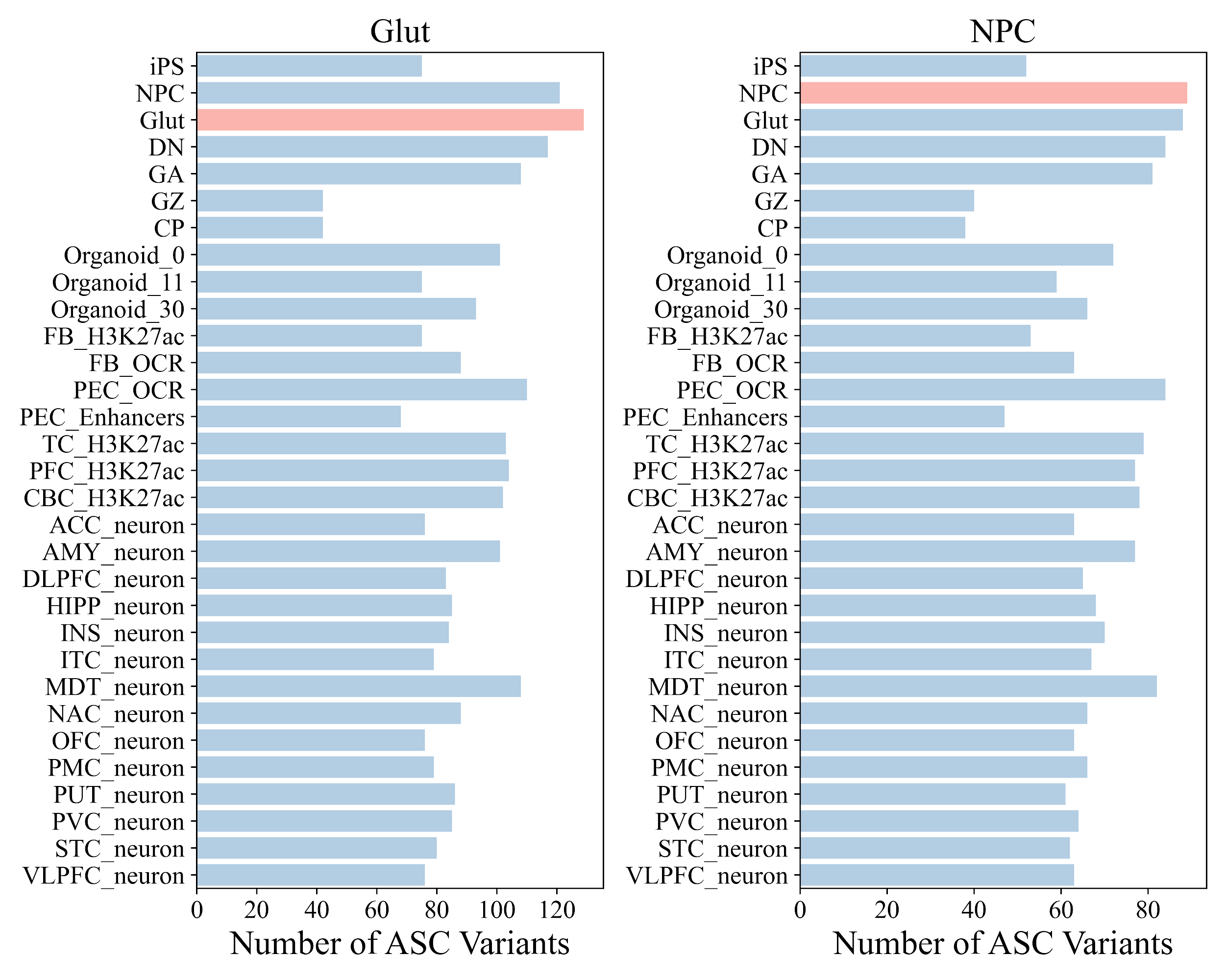


Fig J. Number of experimentally determined ASC variants (two cell types, Glut - left and NPC - right) in top 10,000 MetaChrom predicted functional variants across 31 cell types.


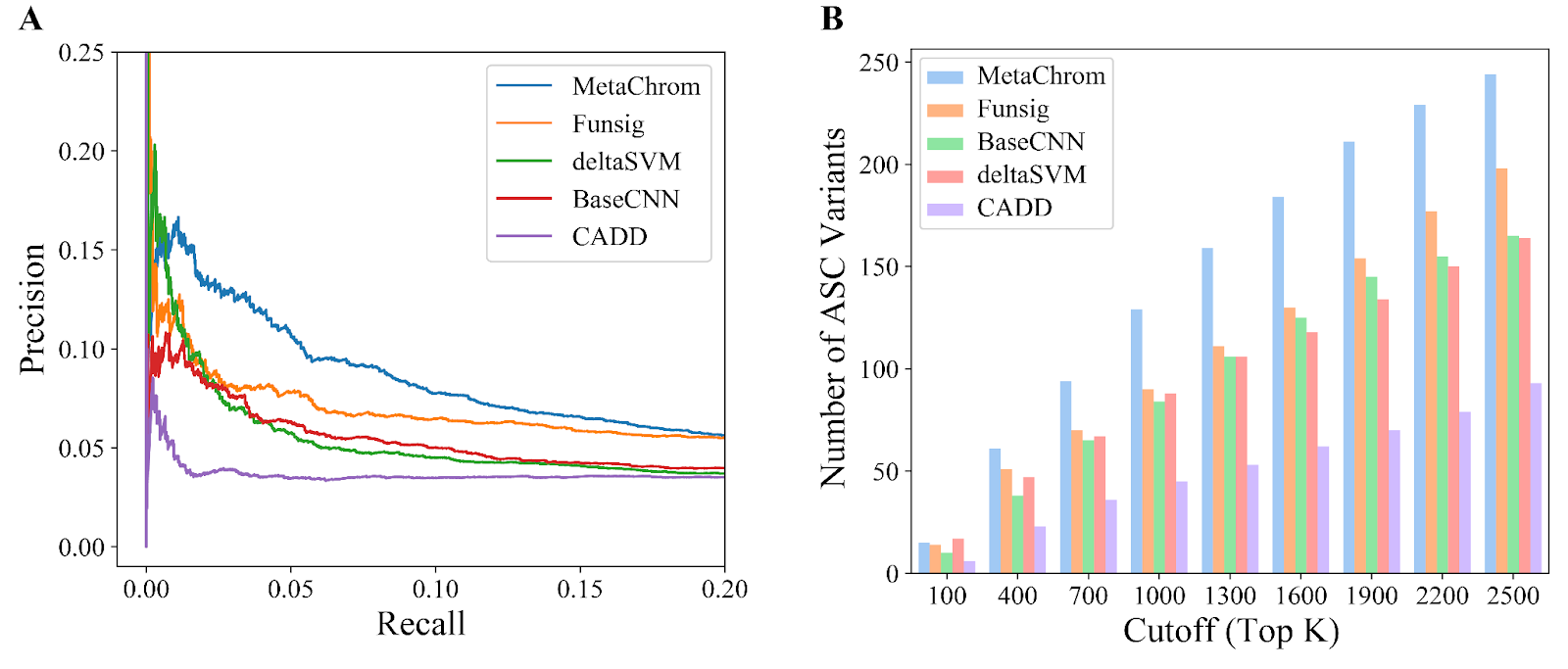


Fig K. Comparison of methods in predicting ASC variants (A) Precision-Recall (PR) curve. We focus on the regime of high-precision and low recall, as in practice, this is where most researchers would be interested in. (B) Number of ASC variants in top K prioritized variants.


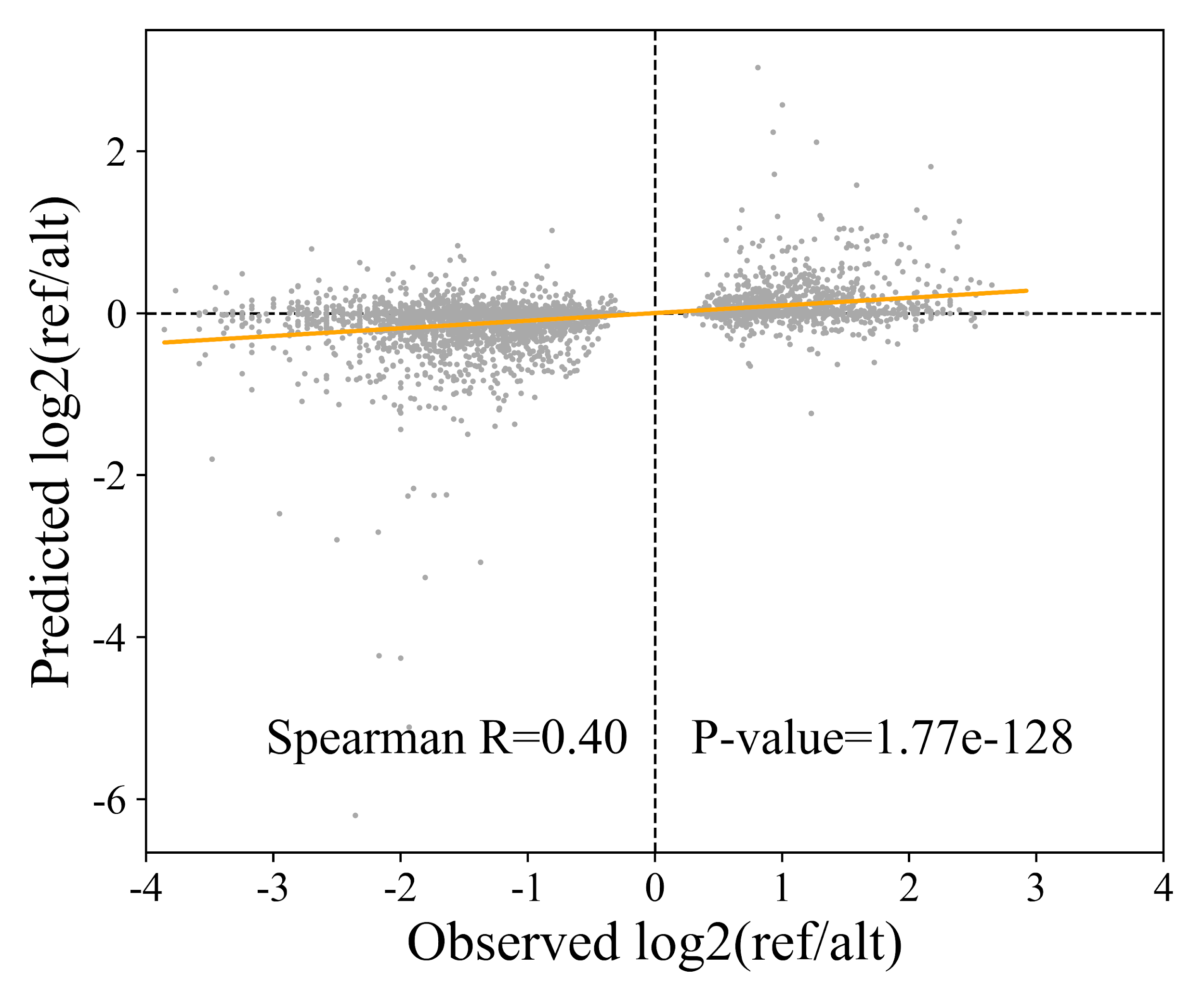


Fig L. The observed allelic imbalance vs. MetaChrom predicted effects on chromatin accessibility of ASC variants in NPC neurons.

Fig M. Likely causal variant rs1080500 and its MetaChrom functional annotations. The candidate SNP rs1080500 is chosen as the reference variant for computing LD and it is highlighted by the red dash line in each panel. The upper panel shows the significance of GWAS SNPs, LD between SNPs and genes in this region. The next panel shows credible set SNPs identified by fine-mapping (PIPs) in this region. The remaining panels show MetaChrom scores in four cell types, two in the fetal stage (FB_OCR and Glut) and two in the adult stage (VLPFC neuron and OFC neuron).
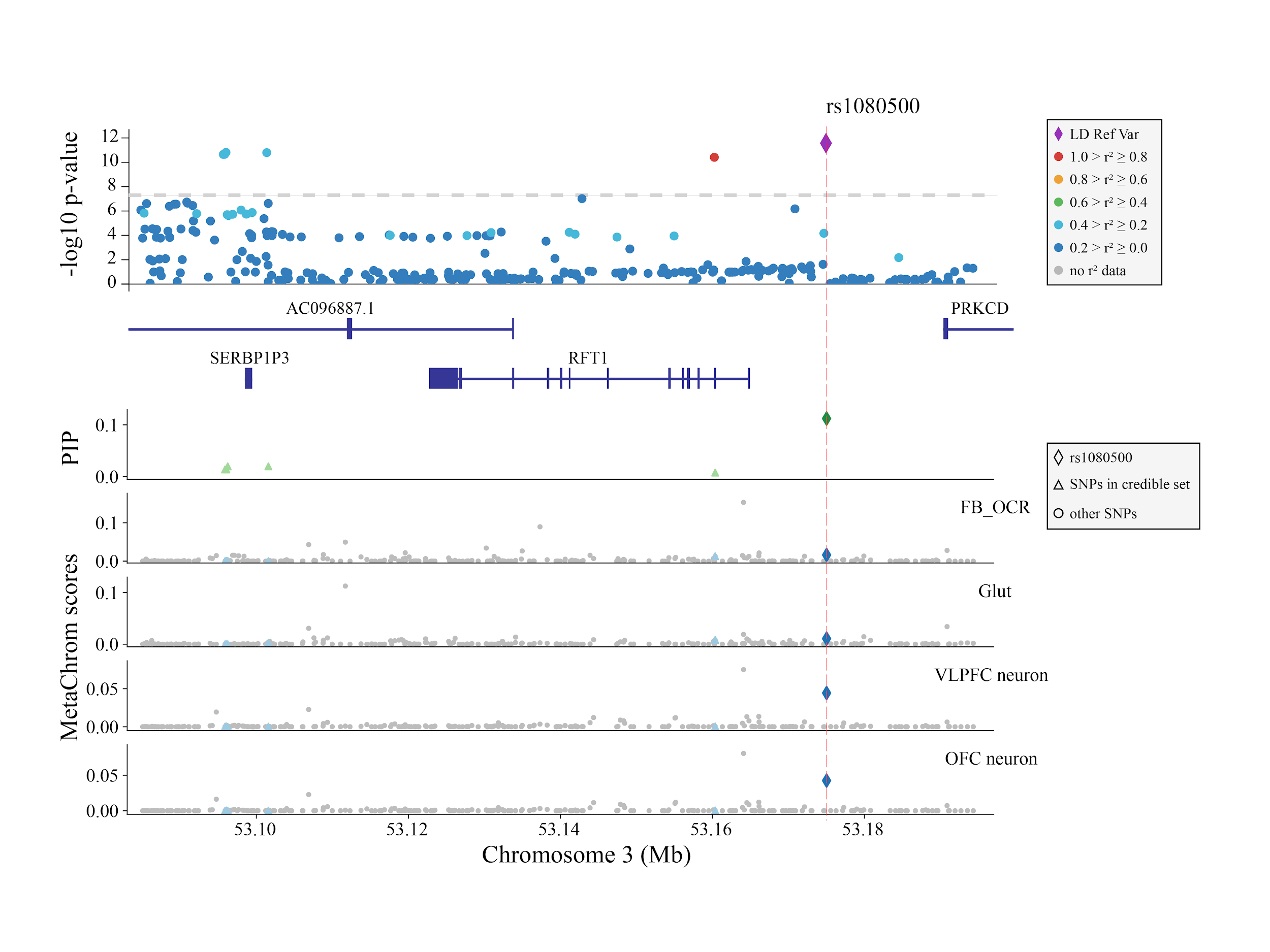


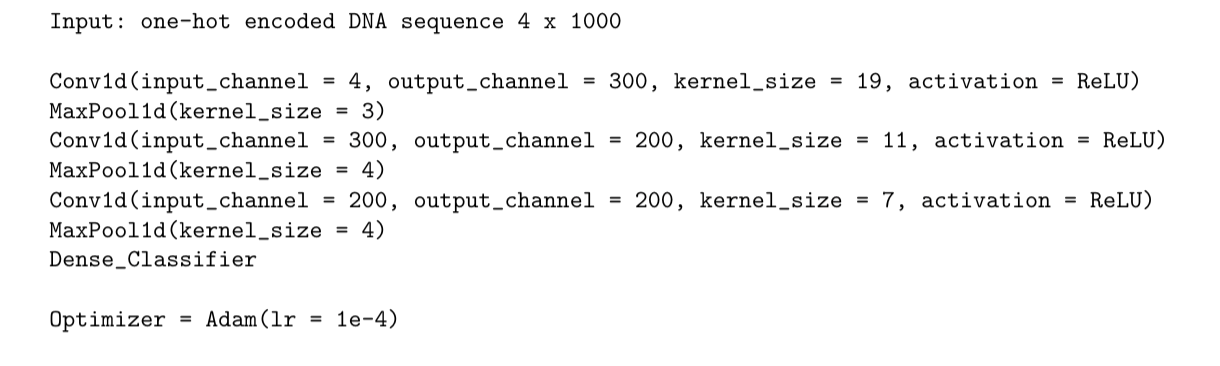


Fig N. Baseline CNN model Architecture
